# Tick-borne infections in human and animal population worldwide

**DOI:** 10.14202/vetworld.2015.301-315

**Published:** 2015-03-12

**Authors:** José Brites-Neto, Keila Maria Roncato Duarte, Thiago Fernandes Martins

**Affiliations:** 1 Department of Public Health, Americana, São Paulo, Brazil; 2 Department of Genetics and Animal Reproduction, Institute of Animal Science, Nova Odessa, São Paulo, Brazil; 3 Department of Preventive Veterinary Medicine, Faculty of Veterinary Medicine and Animal Sciences, University of São Paulo, São Paulo, Brazil

**Keywords:** acarology, argasidae, epidemiology, health public, ixodidae, parasitology veterinary, zoonosis

## Abstract

The abundance and activity of ectoparasites and its hosts are affected by various abiotic factors, such as climate and other organisms (predators, pathogens and competitors) presenting thus multiples forms of association (obligate to facultative, permanent to intermittent and superficial to subcutaneous) developed during long co-evolving processes. Ticks are ectoparasites widespread globally and its eco epidemiology are closely related to the environmental conditions. They are obligatory hematophagous ectoparasites and responsible as vectors or reservoirs at the transmission of pathogenic fungi, protozoa, viruses, rickettsia and others bacteria during their feeding process on the hosts. Ticks constitute the second vector group that transmit the major number of pathogens to humans and play a role primary for animals in the process of diseases transmission. Many studies on bioecology of ticks, considering the information related to their population dynamics, to the host and the environment, comes possible the application and efficiency of tick control measures in the prevention programs of vector-borne diseases. In this review were considered some taxonomic, morphological, epidemiological and clinical fundamental aspects related to the tick-borne infections that affect human and animal populations.

## Introduction

Since the first publish a phylogenetic tree for the ticks inferred from intuition about the relative ‘primitiveness’ of the morphology and life cycles of ticks and their hosts that this knowledge can provide new insights into evolution and studies about the historical biogeography of ticks. Modern methods were applied to the study of the mitochondrial genomes of ticks, providing a framework evolutionary to interpretation the biology and phenotypes of species, aiming to understand the phylogeny and evolution of such organisms [1].

Phylogenetic studies based on morphological analysis and mitochondrial ribosomal DNA sequencing demonstrated that ticks belong to suborders of Arachnida. These ectoparasites belong to the Phylum Arthropoda, Class Arachnida, Subclass Acari, Order Parasitiformes and Suborder Ixodida, sharing the Order Parasitiformes with the suborders Holothyrida, Mesostigmata (commonly known as mites) and Opilioacarida [2].

The origin of ticks was during the pre-mid Cretaceous period (with both the Argasidae and Ixodidae being established in the middle Cretaceous). Ixodids probably evolved from parasites of reptiles in the Paleozoic and Mesozoic era (around 225 million years before the present day). Throughout evolution, ticks lost their primary somatic segmentation presenting tagma as regions of body division. They have a tagma called gnathosoma that includes the mouth pieces that resemble the head of the other arthropods. In the early larval stage of its life cycle, they present only three pairs of legs and when it evolves into an adult form the fourth pair appears. The presence or absence of respiratory spiracles and its position related to the tagma are important to the taxonomic diagnosis [3].

**Figure-1:**
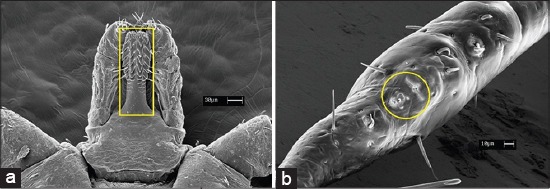
(a) Hypostome presenting reticulate teeth and (b) Haller’s organ. Photo by T.F. Martins.

They differ from the other Acari by presenting a denticulate hypostome; a sensorial organ (Haller’s organ) on the dorsal surface of tarsi in the first pair of legs (Figure-1) and by the absence of claws in the pedipalp. The gnathosoma (capitulum) is the tagma that includes the mouthparts consisting of a pair of pedipalps, with four segments (the third and fourth one are fusioned - tibial-tarsal fusion); a pair of chelicerae and a denticulate hypostome. The pedipalp protects the upper surface of the hypostome and chelicerae. These last one are a very sclerotized structures and used to cut the host during blood feeding allowing the introduction of the hypostome into the skin. Ticks are dioecious species with marked sexual dimorphism and internal fertilization with variations in the mode of transfer of spermatophores. Concerning to embryonic development are oviparous species and some of them exclusively parthenogenetic [4].

Ticks are monophyletic and comprise three families belonging to order Ixodida: Ixodidae Murray, 1844 (known as hard ticks which have a dorsum totally or partially covered with chitin) including 702 described species comprising most of the medical and veterinary importance, containing specimens of the genus *Ixodes*, *Amblyomma*, *Rhipicephalus*, *Dermacentor*, *Haemaphysalis*, *Cosmiomma, Aponomma, Margaropus, Rhipicentor* and *Hyalomma*; Argasidae Canestrini, 1890 (known as soft ticks which have dorsum without chitin) comprising 193 described species containing those from the genus *Argas*, *Ornithodoros*, *Otobius, Antricola, Nothoaspis* and *Carios* and the family Nuttalliellidae including a unique species, *Nuttalliella namaqua* Bedford, 1931 (restricted to South Africa and Tanzania). Various biological differences in lifestyle strategies between hard and soft ticks suggest that blood-feeding adaptations occurred after an evolutive process with significant divergence [5,6].

## General Morphology and Biology of Ticks

### Family Argasidae (Canestrini, 1890)

**Figure-2:**
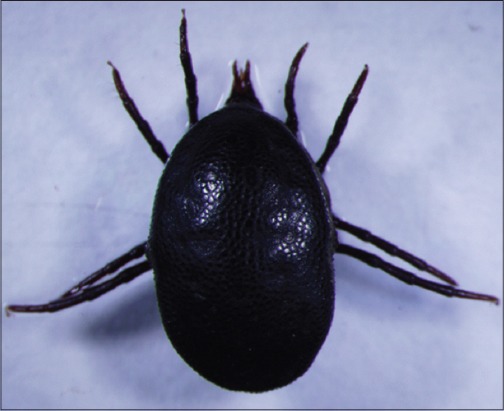
*Nothoaspis amazoniensis*. Courtesy of USP/Marcelo B. Labruna.

**Figure-3:**
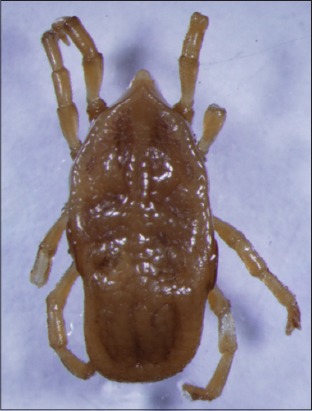
*Carios rondoniensis*. Courtesy of USP/Marcelo B. Labruna.

Their representatives are nidicolous (intimately associated with human and animals) and abundant in arid and semi-arid habitats. Mainly life cycles involve multiple hosts; mating and fecundation occur in the environment, not on the host and perform several oviposition between blood feeding. Among its 193 described species 87 belong to the Neotropical zoogeographic region (Caribbean, South of Mexico and South America), but their phylogeny and taxonomy is as yet controversial, with genus-level classification of the family Argasidae less defined than that in Ixodidae [7]. The Nothoaspinae subfamily is represented by specie *Nothoaspis redelli* Keirans and Clifford, 1975, and a new species *Nothoaspis amazoniensis* Nava, Venzal and Labruna, 2010 (Figure-2). These species of ticks are present in habitats characterized by high temperature and humidity, such as caves colonized by bats and the larvae of *N. amazoniensis* are encountered parasiting *Pteronotus parnellii*, a bat species that is its only apparent host, having rather broad distribution, from central Mexico until to the Amazon and Mato Grosso regions of Brazil. In these caves, another specie of soft ticks, *Carios rondoniensis* Labruna, Terassini, Camargo, Brandão, Ribeiro and Estrada-Peña, 2008 (Figure-3), are also commonly encountered [8]. There are reports about co-extinction associated with its host [9].

**Figure-4:**
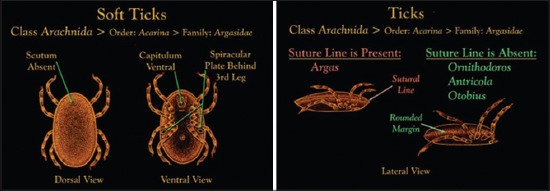
Morphological features of soft ticks demonstrating differentiations between genera. Courtesy of CDC.

Classical argasid tick systematics recognizes five genera, namely *Ornithodoros*, *Antricola*, *Argas*, *Nothoaspis*, and *Otobius* but based on phylogenetic analyses at the generic and subgeneric levels were proposed only four genus to this family: *Argas*, *Carios* (including *Antricola* and *Nothoaspis* species into this genus), *Ornithodoros* and *Otobius*. These representatives have a dorsal-ventral flattened body sharply demarcated from each other by a lateral suture (Figure-4); the gnathosoma is situated in ventral position; there is not a dorsal plate or shield (scutum) as in those of ixodid ticks; and they don’t present eyes [10-12].

**Figure-5:**
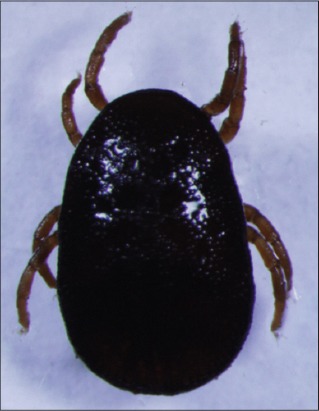
*Argas miniatus*. Courtesy of USP/Marcelo B. Labruna.

Genus *Argas* (Latreille, 1795) is a very common tick in the hen house sucking blood of chickens, turkeys, pigeons and other birds. Facultative autogeny (production of a generation under favorable conditions of temperature and humidity without female feeding) is reported for argasids. In Brazil, the unique existent species is *Argas miniatus* Koch, 1844 (Figure-5), that replaces the cosmopolitan species *Argas persicus* Oken, 1818, which is abundant in rustic hen houses. They are nocturnal, hiding during the day in straw nests, in the crevices of the walls or under the bark of trees. The veterinary and health public importance is due to its irritating biting and hematophagy actions causing a decrease in egg posture and delaying development of new birds, anemia and high mortality in poultry. Furthermore, as vectors they are responsible for transmitting pathogens like *Borrelia* [10,13,14].

**Figure-6:**
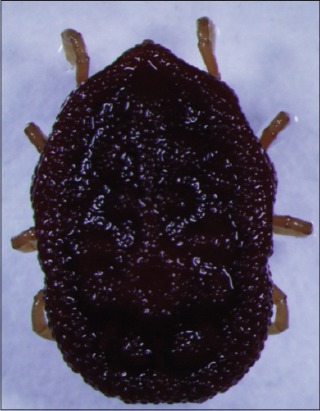
*Ornithodoros rostratus*. Courtesy of USP/Marcelo B. Labruna.

The subfamily Ornithodorinae with Genus *Ornithodoros* (Koch, 1844) comprises 100 species that present a thick body and no sharp boundaries between the dorsal and ventral surfaces. Deep grooves are distributed on the body surface. Eyes can be present in the border of the anterior half of the body. The behavior feeding is diverse, with larvae don’t feeding during this stage and others feeding on quickly. Some species use to remain attach to the host for long periods even days. Facultative autogeny also occurs. *Ornithodoros* are nidicolous ticks living in high-density populations inside to burrows of hosts (endophilic nidicolous) or in harborage in the proximity (non-endophilic nidicolous) being an important relapsing fever disease vector. *Ornithodoros brasiliensis* Aragão, 1923, is an tick found exclusively in the southern Brazilian highlands frequently associated with severe symptoms directly induced by their bite (ticks toxicosis), that may result in clinical signals which ranging from a local pathology (pain and pruritus) to death. For instance, *Ornithodoros rostratus* Aragão, 1911 (Figure-6) is adapted to human habitat and have been found all over Brazilian territory. This argasid lives on the floor of the huts, primitive houses, pigsties and other places where domestic animals live. It is known as “floor-tick” and its bite is very painful and can cause severe local lesions but not seems to transmit pathogens in Brazil [15,16].

**Figure-7:**
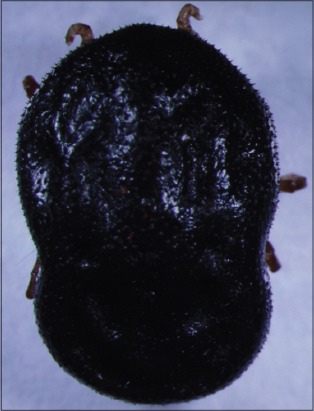
*Otobius megnini*. Courtesy of USP/Marcelo B. Labruna.

Only three species belong to the subfamily Otobinae, Genus *Otobius* (Banks, 1912) presenting a granular integument in the adult stage; absence of eyes. Nymphal stage presents a well-developed hypostome while adults present a vestigial one; adults are not parasites. All the molts occur on the host. The parasitizing stages feed on blood causing irritation and lead to inflammatory injuries. The larval and nymphal stages of *Otobius megnini* Dugès, 1883 (Figure-7), inhabit the ears of equines, cattle, sheep, swines, dogs and even humans (spinose ear tick). The adults live in shelters like branches of trees where they copulate and lay eggs (adults do not feed). Larvae hatch and reach the host where in 5-15 days they molt into the first and second nymphal instars spending up to 6 months in the ear. Then, they leave the host and look for high and dry places to molt into adults. Autogeny is obligatory. This type of parasitism can cause an external parasitic otitis in dogs and human parasitic otitis also were reported [10,13,17].

**Figure-8:**
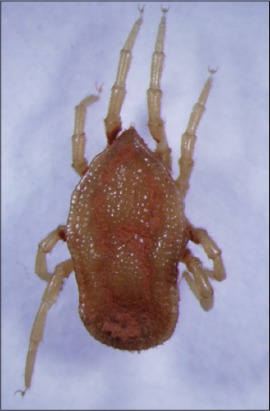
*Antricola guglielmonei*. Courtesy of USP/Marcelo B. Labruna.

The subfamily Antricolinae consists of 17 species belonging to Genus *Antricola* (Cooley and Kohls, 1942). They live in hot and humid caves where bats inhabit, in Neotropical regions, as example of the species *Antricola delacruzi* Estrada-Peña, Barros-Battesti and Venzal, 2004, and *Antricola guglielmonei* Estrada-Peña, Barros-Battesti and Venzal, 2004 (Figure-8). The tick leg ends in a pair of claws with a pulvillus that facilitate climbing walls in caves. They are medium and large sized (females measuring 4-7 mm and males 3-4 mm), with piriform body shape, showing tubercles and well-defined dorsal-marginal grooves. *Antricola* spp. have no functional mouthparts and so they do not feed. Larvae are hematophagous. Autogeny is also obligatory [14,17].

**Figure-9:**
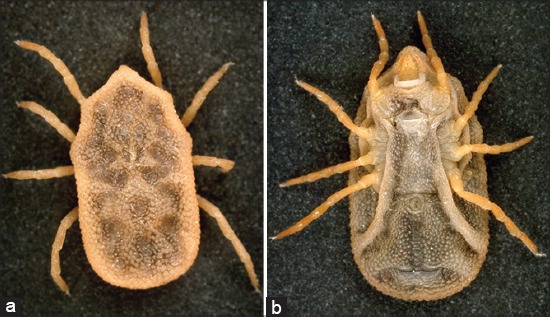
(a) *Carios kelleyi*, dorsal view, (b) *Carios kelleyi*, ventral view. Courtesy of CDC/William L. Nicholson.

The Genus *Carios* (Latreille, 1796) present a species *Carios kelleyi* Colley and Kohls, 1941 (Figure-9) that is very common in the United States (Iowa State) being found in houses or buildings infested by bats. They hide in cracks and crevices and choose to feed on bats; however, if bats abandon or leave a shelter or if there is an increase in bat population for some other reason, ticks can feed on other animals, including humans. The blood feeding is intermitent, and it takes small amounts of blood overnight. To effective infestation, control is important eliminate bats that inhabit households or buildings and also use potential residual acaricides [10,14].

## Family Ixodidae (Murray, 1844)

**Figure-10:**
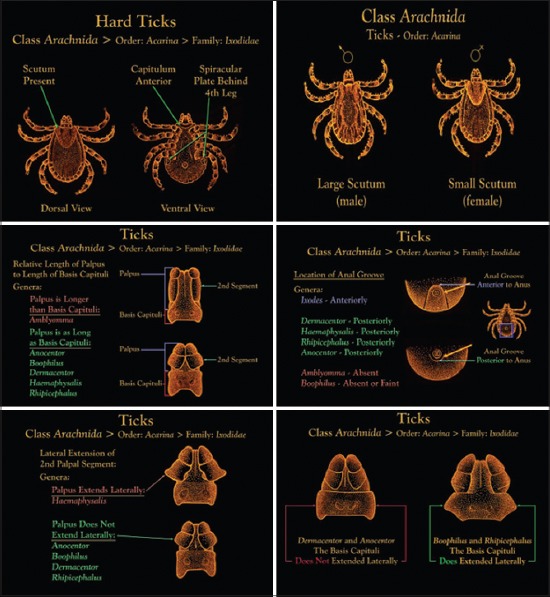
Comparative table of morphological differentiation to genera of hard ticks. Courtesy of CDC.

This family comprises the known “hard ticks” because they present a large anterodorsal sclerite, a dorsal plate or scutum. There is a remarkable sexual dimorfism, and the posterior border of opistosome can be divided into sclerotized structures called festoons (Figure-10). Ixodid or hard ticks (Acari: Ixodida: Ixodidae) are blood-feeding ectoparasites and parasitize several hosts (life cycles envolving 1-3 hosts) and presenting free-living stages in the environment, being capable of transmitting a broad range of human and animal pathogens in worldwide. They are relatively large (2-20 mm) [2,10]. This family comprises the major number of genera (14) and includes 702 species in which 120 are described from Neotropical region distributed in two major groups and 5 subfamilies: Prostriata group, subfamily Ixodinae consisting in a single genus, *Ixodes*, with 243 described species; Metastriata group including subfamilies Amblyomminae (2 genera: *Amblyomma* and *Aponomma*: 130 species); Haemaphysalinae (1 genus, *Haemaphysalis*: 166 species); Hyalomminae (1 genus, *Hyalomma*: 27 species) and Riphicephalinae (seven genera: *Dermacentor*, *Cosmiomma*, *Margaropus*, *Nosomma*, *Anomalohimalaya*, *Rhipicentor* and *Rhipicephalus Boophilus* comprising in total 136 described species). Molecular markers have been developed and applied specifically to study those ticks, and associated with the conventional technics, the evidences demonstrate that part of Amblyomminae (species previously considered as the genus *Aponomma*) now belong to basal Metastriata, subfamily Bothriocrotinae and Hyalomminae belong to Rhipicephalinae [18,19].

**Figure-11:**
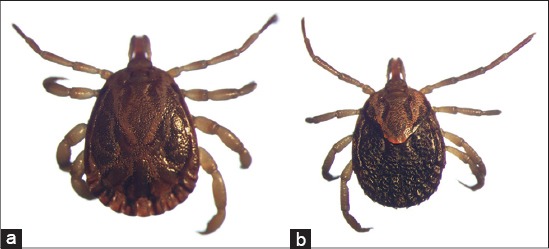
(a) *Amblyomma cajennense sensu lato* ♂ and (b) *Amblyomma cajennense sensu lato* ♀. Courtesy of USP/Marcelo B. Labruna.

The Genus *Amblyomma* (Kock, 1844) occurs worldwide, in all continents, except Antartic, ranging between parallels latitude 40°N and 40°S. There are 106 described species, from which half one are found in the American continent. In the Neotropical zoogeographic region 57 species are known. This genus parasitize a large variety of hosts, most of them from Order Mammalia. Birds are rarely parasitized by adult ticks, but often they host immature stages of some species. Amphibians and reptiles also are potential hosts. In the neotropics, they constitute a major public health problem because the high prevalence of human cases of parasitism and the excellent potencial as vectors for transmitting infectious agents. *Amblyomma* species have a three-host life cycle, with rare exceptions (*Amblyomma rotundatum* Koch, 1844). They belong to Metastriata group in which the anal groove is in posterior position in relation to the anal pore. They have an ornatedorsal plate (scutum or shield); they have eyes, long pedipalps and hypostome and spiracular plate-comma shaped. The species identification is carried out by the use of five currently available dichotomous keys specifically applied to this genus [2,13]. *Amblyomma cajennense* Fabricius, 1787 (Figure-11) is considered a tick widely distributed in the Neotropical regions and the most widespread specie in the American continent from Southern Texas (USA) to northern Argentina, in environments very diverse as the dry grasslands, the highlands and the tropical forest. Have low host specificity and a three-host life cycle characterized by one generation per year and feeding on a variety of vertebrate hosts, mostly mammals (such as the horse and the capybara). Recent studies for reassessment of the taxonomic status of *A. cajennense* showed that morphological analyses were congruent with the results of the molecular and biological analyses, through of mitochondrial and nuclear genetic evidences [20] indicating that *A. cajennense* is a complex of 6 species, through the redescription of *A. cajennense* sensu stricto Fabricius, 1787 (found in the Amazonian region of South America), the description and definition of 3 new species, *Amblyomma tonelliae* Nava, Beati and Labruna, 2014 (associated with the dry areas of central-northern Argentina until to Bolivia and Paraguay), *Amblyomma interandinum* Beati, Nava, and Cáceres, 2014 (reported from the northern Peru) and *Amblyomma patinoi* Labruna, Nava and Beati, 2014 (occuring in the Eastern Colombia) and validation of 2 species that were synonymy in the past, *Amblyomma mixtum* Koch, 1844 (present from Texas, U.S.A. to western Ecuador) and *Amblyomma sculptum* Berlese, 1888 (distributed from the humid areas of northern Argentina, to the contiguous regions of Bolivia and Paraguay and the coastal and central-western states of Brazil) [21]. The existence of six clades corresponding to different ecological features [20] and studies of the abiotic variables (temperature, humidity and seasonal patterns) would provide a better understanding of the abiotic preferences of the species in the *A. cajennense complex* and its respective niches. These studies have shown that, *A. mixtum*, *A. cajennense*, *A. tonelliae* and *A. sculptum* come presenting allopatric distributions in much of its home range, with geographically isolated subpopulations, but also come occurring a parapatric speciation with adjacent distributions between at least two divergent species, *A. sculptum* and *A. tonelliae* [22].

**Figure-12:**
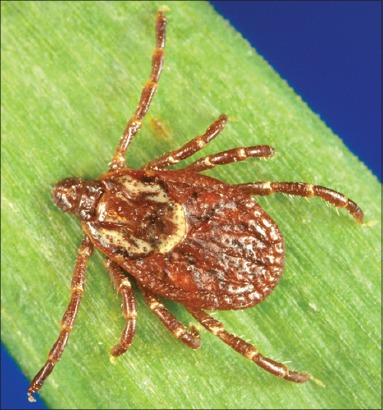
(a) *Rhipicephalus (Boophilus) microplus* ♂ and (b) *Rhipicephalus (Boophilus) microplus* ♀. Courtesy of USP/Marcelo B. Labruna.

**Figure-13:**
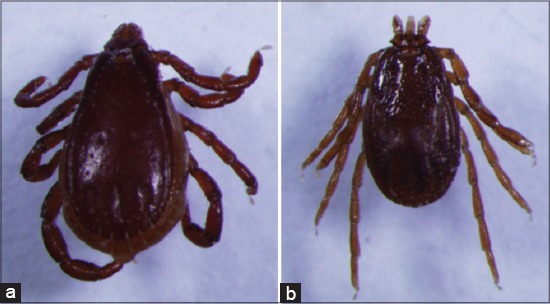
(a) *Rhipicephalus sanguineus* sensu lato ♂ and (b) *Rhipicephalus sanguineus* sensu lato ♀ Courtesy of USP/Marcelo B. Labruna.

Originated from Africa the Genus *Rhipicephalus* (Kock, 1844) is consisted by a little more than 70 species in the world. Molecular philogeny studies show evidences that this genus comprises the five *Boophilus* species that are now considered a subgenus of *Rhipicephalus*. All species are brown or reddish brown colored, it has not ornate shield, it has eyes, short palps and hypostome, and present a hexagonal basis capituli. Males present two to four adanal plates, and it can present, in some species a posterior appendage. The majority of its representatives have three-host life cycle, and some of them have one or two-host life cycle. The specie *Rhipicephalus (Boophilus) microplus* Canestrini, 1887 (Figure-12) is a cattle parasite but also can be found in other domestic or wild animals. It is a typical one-host life cycle tick. Its distribution range cover the inter-tropical zone in Americas (although its is erradicated in North America), Africa, Oceania and it was introduced in Brazil with the cattle by colonizers [23]. *Rhipicephalus (Boophilus) australis* Fuller, 1899 (Australian cattle tick) is a one-host tick with a monotropic type of behaviour, where the time spent by the three life-stages on the host is about 3 weeks and egg laying can be completed in about 4 weeks. *Rhipicephalus australis* may transmit the protozoans *Babesia bovis* and *Babesia bigemina* in the bovine babesiosis, the bacterium *Anaplasma marginale* in the anaplasmosis and *Borrelia theileri* in the spirochaetosis in cattle [13]. The brown dog tick, *Rhipicephalus sanguineus* sensu lato Latreille, 1806 (Figure-13), is a three-host cosmopolitan tick species (each life stage requires a new host to feed on), parasiting dogs and other hosts, including other mammals, birds and humans in tropical and temperate regions, being considered an urban plague very widespread, and presenting a profile ethological by nature endophilic (adapted to indoor living) and monotropic (all developmental stages feed on the same host species)[24]. Currently eleven ticks species are classified within the “*R. sanguineus*” species complex: *R. sanguineus* Latreille, 1806 (distributed worldwide, especially in tropical and subtropical regions, strongly associated with the Canidae, mainly endophilic, but also showing harborage nidicolous behaviour, occasionally exophilic), *Rhipicephalus bergeoni* Morel and Balis, 1976 (mainly a parasite of Artiodactyla, Bovidae, probably restricted to Sudan and Ethiopia and exophilic), *Rhipicephalus camicasi* Morel, Mouchet and Rodhain, 1976 (parasite of Bovidae and Camelidae in northern Africa, Egypt, Sudan, Ethiopia, Somalia, probably Kenya, in coexistence with *R. sanguineus*, probably exophilic), *Rhipicephalus guilhoni* Morel and Vassiliades, 1963 (parasite of Bovidae and Muridae, probably restricted to Sudan and Ethiopia, adults exophilic), *Rhipicephalus leporis* Pomerantsev, 1946 (mainly parasite of Leporidae and Canidae, Central Asia, east to Caspian sea, endophilic), *Rhipicephalus moucheti* Morel, 1964 (mainly parasite of Canidae, Western Africa, exophilic), *Rhipicephalus pumilio* Schulze, 1935 (reported from several Orders of Mammalia and Aves, reported from humans, Central Asia, probably restricted to endophilous habitats), *Rhipicephalus pusillus* Gil Collado, 1938 (restricted to the European rabbit, *Oryctolagus cuniculus* and found only in the western Mediterranean, completely endophilic), *Rhipicephalus schulzei* Olenev, 1929 (mainly parasite of Rodentia and Lagomorpha, reported from humans, Central Asia, endophilic), *Rhipicephalus sulcatus* Neumann, 1908 (parasite of several orders of Mammalia, parts of central and southern Africa, Zimbabwe, Botswana, probably introduced into western Africa, exophilic) and *Rhipicephalus turanicus* Pomerantsev, 1936 [25]. It spreads by dwellings climbing the walls (negative geotropism) hiding in crevices and roofs, being highly reproductive and very hard to control the infestations in that all molts occur in the environment and not on host. Females lay 2000-3000 eggs in their entire lives. Unfed larvae can survive up to 8½ months, nymphs up to 6 months and adults up to 19 months. They attack any region of the host body being more frequent in the anterior limbs and in the ears causing irritation, disconfort and dermatitis. *R. sanguineus* sensu lato is capable of transmitting many zoonotic agents, such as tick-borne pathogens (*Rickettsia rickettsii*, a spotted fever agent in humans and *Rickettsia conorii*, a mediterranean spotted fever agent), *Babesia vogeli* (canine babesiosis agent), *Hepatozoon canis* and *Ehrlichia canis* (canine monocytic ehrlichiosis agent) [26].

**Figure-14:**
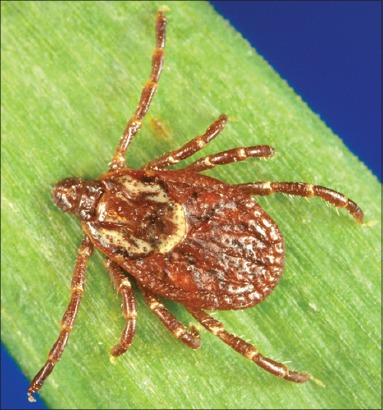
*Dermacentor variabilis* ♀. Courtesy of CDC/DVBID/Gary O. Maupin.

Eight species are described to the Neotropical region in the Genus *Dermacentor* (Kock, 1844). The most has a three-host life cycle, excepting *Dermacentor albipictus* Packard, 1869 and *Dermacentor nitens* Neumann, 1897, which are one-host ticks. They present a dorsal basis capituli quadrangular shapped, eyes and seven to eleven festoons. *D. nitens* feeds on equine: Horses, donkeys and mules. It is found parasitizing the pinna (ear canal) and nasal diverticulum causing supuration, secondary infections and myiasis. Females lay an average of 3000 eggs in the ground. Larvae can withstand periods of starvation up to 71 days. Its is a one-host tick. *D. nitens* is one of the main vector of *Babesia equi* and *Babesia caballi*. *Dermacentor variabilis* Say, 1821, is the principal vector of Rocky Mountain Spotted Fever rickettsiae (American Dog Tick; Figure-14). It causes tick paralysis and transmits Tularemia. *Dermacentor andersoni* Stiles, 1908, is also vector of Rocky Mountain Spotted Fever rickettsiae (Rocky Mountain Wood Tick)[27].

**Figure-15:**
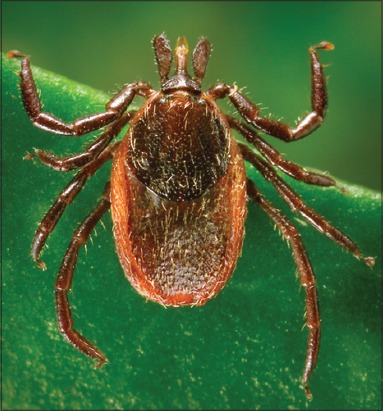
*Ixodes pacificus* ♀. Courtesy of CDC/William L. Nicholson.

**Figure-16:**
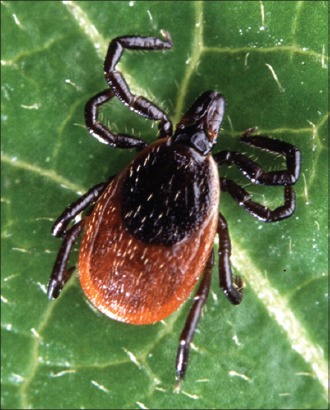
*Ixodes scapularis* ♀. Courtesy of USDA/Scott Bauer.

The Genus *Ixodes* (Latreille, 1795) is the most important American and European tick, vector for *Borrelia burgdorferi* which causes the Lyme disease. The principal species are *Ixodes pacificus* Cooley and Kohls, 1943 (Figure-15), *Ixodes scapularis* Say, 1821 (Figure-16) and *Ixodes hexagonus* Leach, 1815. Another species are also envolved on transmission of tick-borne diseases: *Ixodes cookei* Packard, 1869 (Powassan encephalitis virus), *Ixodes holocyclus* Neumann, 1899 (Australian paralysis tick in domestic animals, wildlife and humans by holocyclotoxin), *Ixodes ovatus* Neumann, 1899 (*Rickettsia japonica*), *Ixodes persulcatus* Schulze, 1930 (Tick-borne encephalitis virus [TBEV]), *Ixodes ricinus* Linnaeus, 1758 (Louping ill virus in sheep, babesiosis in cattle, human babesiosis, and human granulocytic ehrlichiosis) and *Ixodes rubicundus* Neumann, 1904 (Tick paralysis in sheep by Karoo paralysis toxin) [13,28].

## Medical and Veterinary Importance

The problem of ticks and tick-borne diseases in urban areas has attracted attention of researchers for several decades, and new data concerning the occurrence of ticks and recognition of zoonoses in urban areas are continuously studied. The conditions for the persistence of ticks population in the urban environment are relationated to existence of urban areas potentially suitable for tick proliferation, such as urban forests, private gardens, public woods and riparian forests into river banks. The successful survival of ticks depends on optimal temperature and humidity, that are the main components of microclimate, in their habitats and the availability of appropriate tick hosts into an urban locality suitable to tick life [29]. Recent studies confirmed that ticks that inhabit urban localities originate from tick populations persisting in wild natural habitats around cities and towns, and environmental conditions in urban localities correspond to those under which tick populations live in wild nature, the more suitable for ticks these localities are [30,31].

Ectoparasites and its hosts do not exist in isolation being its abundance and activity affected by various abiotic factors, such as climate and other organisms (predators, pathogens, and competitors) presenting thus multiples forms of association (obligate to facultative, permanent to intermittent and superficial to subcutaneous) developed during long co-evolving processes. Ticks are widespread globally, and its dynamics is closely related to the environmental conditions [32].

Ticks can transmit pathogens by the saliva that is the main pathway by which microorganisms agents achieve the host’s bloodstream constituting the second vector group that transmits the major number of pathogens to humans, being surpassed only by culicids mosquitos [33], however play a role of important primary for animals in the process of diseases transmission. They are obligatory hematophagous ectoparasites of vertebrates and are responsible for vectors or reservoirs in the transmission of pathogenic fungi (*Dermatophilosis*), protozoa (*Babesiosis* and *Theileriosis*), viruses (Crimean–Congo hemorrhagic fever, Powassan encephalitis, Kyasanur forest disease (KFD), Louping ill, Colorado tick fever, Omsk hemorrhagic fever (OHF), West nile virus (WNV), African swine fever virus (ASFV) and others tick-borne encephalitis (TBE), rickettsial (Rocky Mountain spotted fever, Brazilian spotted fever, Boutonneuse Fever or Mediterranean spotted fever, African tick bite fever, Cowdriosis, Human granulocytic anaplasmosis, Human monocytic ehrlichiosis, others ehrlichiosis and anaplasmosis) and others bacteria (lyme disease, tularemia, Q fever, relapsing fever and borreliosis) during their feeding process on the hosts [34].

During feeding, ticks can lead to several deleterious effects on the host such as traumatic action by tick bites determining a dermatoid process with direct damage to the skin and other subcutaneous tissues, inflammation and significant blood loss by hematophagism. It can be observed compression and laceration of cells and tissues, generally associated with pruritis, edema, erythema, excoriation, papules, lichenification, scaling and ulceration by self-trauma. Predisposition to secundary process for bacterial infection can stimulate immune responses in some individuals leading to hypersensitivity. Apart from causing diseases, the behavior of ticks also may cause indirectly harm, causing disturbance, increasing levels of stress and in cattle, for example, leather depreciation, being economically the most important ectoparasites of cattle. Direct effects of tick infestation on cattle include the sucking of blood which causes anaemia and damage to the skin or hide, with downstream effects resulting in substantial losses in terms of reductions in productivity and fertility, body weight, and milk production, and in toxicoses, paralysis, and mortality [35].

Furthermore, ticks can induce a motor illness causing an acute, ascending flaccid paralysis with a rapid clinical evolution and lethal if not immediately treated both in humans and in animals. It is mainly related to ixodid ticks by existent neurotoxins in the females saliva. Some species (*D. andersoni*, *D. variabilis*, *I. holocyclus* and *Amblyomma* spp.) produce paralyzing toxins that act decreasing acetylcholine levels damaging local neuron synapses by blocking the neuro-muscular junctions. 5 or 7 days after a tick attaching the pacient present the first symptoms feelling fatigue, numbness in the legs and muscle aches, with development of flaccid paralysis. The clinical framework can be complicated with difficulty swallowing, facial paralysis, convulsions and death by respiratory arrest [36].

Soft ticks also may transmit rickettsia and some protozoan and filarial species, important to human and animal health, such as, *Aegyptianella pullorum* (Aegyptianellosis - *Argas* spp.), *Wolbachia persica* (*Argas arboreus*), *Rahnella aquatilis*, *Pseudomonas fluorescens*, *Enterobacter cloacae*, *Chryseomonas luteola* and *Chryseobacterium meningosepticum* (*A. persicus*), *B. burgdorferi* (Lyme disease - *Argas vespertilionis*), *Rickettsia scc3*, *Rickettsia hoogstraalii*, *Rickettsia felis* and *Rickettsia* spp. (Spotted fever - *Carios capensis*, *C. kelleyi*, *Carios sawaii* and *Ornithodoros moubata*), *Borrelia* spp. and *Borrelia lonestari* (*C. kelleyi*), *Bartonella henselae* (Cat scratch disease – *C. kelleyi*), Deltaproteobacteria (Bovine Epizootic Abortion - *Ornithodoros coriaceus*), *Babesia meri* (*Ornithodoros erraticus*), *Coxiella burnetii* (Q fever - *Ornithodoros lahorensis*, *Ornithodoros moubata* and *Ornithodoros sonrai*), *B. equi* and *Acanthocheilonema viteae* (*O. moubata*) and *Dipetalonema viteae* (*Ornithodoros tartakowskyi*)[37].

On the medical and veterinarian point of view, most of 100 arthropod-borne infections can be associated with 116 tick species (32 argasid species and 84 ixodids). Tick-borne diseases are common in the medical and veterinary clinical settings alerting to the importance of a one health approach to these diseases, unifying physicians and veterinarians in their syndromic surveillance for the management of zoonoses and communicable diseases common to man and animals [38].

## Tick-borne Viruses

All tick-borne virus are arboviruses and its survival depends on the infection and replication in the ticks and vertebrate host cells, establishing one association between a tick-borne virus and its tick vector species very intimate and highly specific. They are established in six different virus families: Asfarviridae, reoviridae, rhabdoviridae, orthomyxoviridae, bunyaviridae and flaviviridae. The viruses are obligate parasites like the ticks, and while ticks need a blood meal from their hosts to survive, the viruses require the transcription, translation, and post-translation processing machinery of their hosts to propagate [39].

The TBEV belongs to genus *Flavivirus* and it is the most important human arboviral pathogen in Europe being subdivided into three sub-types, distributed by their principal vector species, *I. persulcatus* and *Haemaphysalis concinna* for Far-Eastern subtypes and Siberian subtypes, *I. ricinus* and *Haemaphysalis punctata* for the Western European subtype. Vectors of TBEV are characterized by a life cycle in what the virus can be kept infecting during all four developmental stages (eggs, larvae, nymphs and adults), surviving during metamorphosis from larva to nymph and nymph to adult (trans-stadial survival) being a key to maintaining the transmission cycle of TBEV, particularly in three-host ticks, indicating that ticks are long-term reservoirs that have shaped the evolution and pathogenic properties of TBEV, although wild rodents are frequently referred to as the reservoirs for this virus [40]. TBE viruses can cause human disease with clinical signals associated with development of inflammatory disease of the central nervous system (CNS), coming form a simple benign meningitis up to a meningoencephalomyelitis with fatal outcome (death rate 20%). In animals, it were detected in monkeys, dogs and horses with neurological clinical disorders and the goats, sheep and cows infected by ticks transmitting infectious virus by their milk to their offspring and also to humans [41].

KFD and OHF are caused by RNA genome virus belonging to the genus *Flavivirus* on the family flaviviridae. KFD determines an incidence of cases of hemorragic fevers in India where rodents, shrews, and monkeys are common hosts for the virus that also can cause epizootics with high fatality in primates and humans that inhabit endemic areas that are accidentally infected. The KFD vector is the nymphal stage of *Haemaphysalis spinigera* and *Haemaphysalis turturis* [42]. OHF is an acute viral disease prevalent in the lacustrine region of Western Siberia (Omsk, Novosibirsk, Kurgan and Tyumen) in Russia, where rodents serves as the primary host and tick vectors include *Dermacentor reticulatus*, *Dermacentor marginatus*, *Dermacentor pictus* and *I. persulcatus*. The disease in humans presents clinical signs of high fever, headache, nausea, oral vesicular lesions, severe muscle pain, cough and prostration during 1-2 weeks, with possible worsening of the condition in a third of patients characterized by pneumonia, severe haemorrhagic manifestations of respiratory, intestinal and reproductive system, nephrosis, meningitis, or a combination of these complications, determining a mortality between 2% and 10% [43].

WNV is a member of the genus *Flavivirus* isolated from species of mosquito in the United States and isolated from haematophagous species ectoparasites of birds (hard ticks, *I. ricinus* and *I. scapularis* and soft ticks, *O. moubata* and *C. capensis*) in regions of Europe, Africa and Asia where WNV is a endemic disease causing clinical neurological signs and encephalomyelitis into numerous birds and mammals species. Although, mosquitoes are the primary vectors and the ticks are not efficient vectors of WNV, the virus can persist for a comparatively long time in infected ticks and it can be transmitted between vertebrate hosts suggesting a reservoir potential for WNV [44,45].

Colorado tick fever is caused by a stranded RNA virus genome, with 12 segments, belonging to *Coltivirus* genera, family Reoviridae, originally found in the United States and Canada (Western North America) with the wood tick *D. andersoni* acting as vector and wild mammals as reservoirs at the transmission of this zoonosis characterized by clinical signs of fever, headache and myalgia with benign evolution, and that may be associated with conjunctivitis, rash and meningitis [46].

Two arthropod-borne viruses (Congo and Crimea hemorragic fever) are notified in Africa, from western China through southern Asia and the Middle East to southeastern Europe resulting in high mortality. The etiological agent is a RNA genome virus, genus *Nairovirus*, Bunyaviridae family transmitted in nature by ticks, *Amblyomma variegatum*, *Hyalomma anatolicum*, *Hyalomma marginatum*, *H. marginatum rufipes*, *Hyalomma truncatum, Hyalomma lusitanicum, Hyalomma plumbeum, Rhipicephalus bursa, Rhipicephalus rossicus* and *D. marginatus* [47]. The incubation period in humans lasts between 5 and 12 days from the tick bite to onset of symptoms of the disease with high fever, chills, headache, dizziness, diffuse myalgia, abdominal pain, nausea, vomiting, diarrhea, hyperemia and edema of the face and neck, conjunctival congestion and bradycardia. Hemorrhagic manifestations include epistaxis, gingival bleeding, bleeding gastric mucosa and hematuria, with death due to shock from blood loss, neurological complications, pulmonary hemorrhage or intercurrent infections resulting in a rate of approximately 30% lethality [48].

The Powassan Encephalitis is a disease whose causative agent is a RNA genome virus of the genus *Flavivirus* on the family Flaviviridae, diagnosed in the Northern Hemisphere, including Canada, United States and Russia, transmited by ticks *D. andersoni, D. variabilis, Ixodes cookie* and *I. scapularis* [49]. The Powassan Encephalitis virus incubation period ranged from 8 to 34 days and symptoms observed in humans are characterized by a clinical picture with fever, headache, disorientation, vomiting, prostration, respiratory distress, and spastic paresis. The infection can lead to meningoencephalitis and a meningitis. The disease in animals is a subclinical infection, but wild animals and horses can producing a viremia with development of neurological signs [50].

Louping ill is a virus caused by the RNA genome virus of the genus *Flavivirus* on the family Flaviviridae belonging to the complex of viruses transmitted by tick *I. ricinus* in Western Europe. The incubation period in humans is 2-8 days. The disease occurs in a biphasic manner. The first phase is characterized by fever, retro-orbital pain, and headache; in the second phase is characterized by nervous symptomatology, in the form of meningoencephalitis or paralytic poliomyelitis. In enzootic areas, animal disease affects sheep and cattle, after one incubation period of 6-18 days with a febrile and viremic form, or the virus may invade the CNS and cause encephalomyelitis. The most prominent symptoms are fever, motor incoordination, tremors, salivation and lethargy [51].

The ASFV is caused by a deoxyribonucleic acid (DNA) genome arbovirus belonging to the asfarviridae family, genus *Asfivirus*, that affects only swine species and causes a haemorrhagic fever highly lethal to pigs, being one of the most important viral diseases included in the A list of the OIE. In nature, ASFV circulates in two types of enzootic cycles (sylvatic and domestic), both of which involve porcine hosts and Argasid ticks of the genus *Ornithodoros*, including the species *O. moubata*, *O. porcinus*, *O. savignyi*, and *O. sonrai*, in the Africa; members of complex *O. erraticus*, on the Iberian Peninsula, Caucasus countries and the Russian Federation; and *O. coriaceus*, *O. turicata*, *O. parkeri* and *O. puertoricensis*, in North America and the Caribbean [52].

## Tick-Borne Rickettisae

The global distribution of tick-borne rickettsial and the tick population’s expansion can introduce rickettsial agents to new geographic areas according to the density and distribution of the predominant tick vectors and the population density of reservoir hosts establishing novel relationships on the dynamic between these pathogens and vectors [53].

The boutonneuse fever or mediterranean spotted fever is a ganglionic-cutaneous rickettsiosis caused by *R. conorii* in Africa, Asia and Europe, transmitted primarily by *R. sanguineus* sensu lato, taking the dog as a reservoir but also presenting second specie of vector, *R. turanicus*. Clinically it is characterized by a hardened initial lesion with a necrotic center on the site of the tick bite. The lesion evolves to ulceration with an inflammatory halo known as scab or black spot. Symptoms are followed by fever, severe headache and muscle and joint pain, lymphadenopathy, general discomfort and a generalized maculopapular rash that appears between the 4^th^ and 5^th^ day of fever and lasts about a week [54].

Anaplasmosis is an acute, subacute or cronical tick-born illness from cattle worldwide caused by a punctiform pathogen that is located at the periphery of the erythrocytes, described as *A. marginale*. Infected cattle can present clinical signs characterized by fever, apathy, anorexia, weakness, weight loss, tachycardia, tachypnea, and ruminal atony with 30-50% mortality rate. The vector is *Rhipicephalus (Boophilus) microplus*. Human granulocytic anaplasmosis is caused by *Anaplasma phagocytophilum* and transmitted by ticks *H. concinna*, *H. punctata*, *I. ricinus*, *I. pacificus*, *I. scapularis* and *R. bursa* in Europe and North America. Human fatalities occur in <1% of cases associated with opportunistic infections, after clinical evidences of fever, headache, lethargy, myalgia, elevated liver function enzymes and reduced platelets [55].

The heartwater or Cowdriosis has been reported in South Africa and sub-Saharan Africa but also in some Caribean regions. There are about ten different *Amblyomma* African species that can act as a vector, and the more implicated are *A. variegatum*, *Amblyomma hebraeum* and *A. pomposum*. These rickettsiosis are caused by *Ehrlichia* (*Cowdria) ruminantium* (Order Rickettsiales; Family Anaplasmataceae) which is an obligately intracellular proteobacterium transmitted through all *Amblyomma* instars, determining a disease in some wild and all domestic ruminants. It is characterized by an enzootic disease in sheep, goats and camels leading to high morbidity and mortality. Due to the recent introduction of *A. variegatum* in Caribean there is a concern that the disease can spread throughout the American continent by the vector dispersion involving migratory birds. It is a febrile disease of ruminants with an incubation period of 2-3 weeks and clinic signs of loss of appetite, lethargy, diarrhea, dyspnoea indicative of pulmonary edema, neurological signs of restlessness, walking in circles, muscle tremors, aggressive or anxious behavior, opisthotonos and nystagmus, with fatal outcome. The disease has a significant economic importance in livestock with mortality rates due to heartwater from 20% to 90% in susceptible ruminants [56,57].

African tick bite fever is a disease of benign evolution caused by *Rickettsia africae* and transmitted by ticks *A. hebraeum* and *A. variegatum* to human mainly on South Africa (80%) and West India with clinical signals of mild fever, multiple inoculation eschars and a vesicular rash followed by the enlargment of lymph nodes draining the eschared area [58].

**Figure-17:**
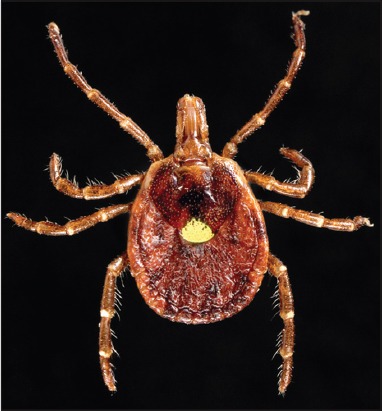
*Amblyomma americanum* ♀. Courtesy of CDC/William L. Nicholson.

**Figure-18:**
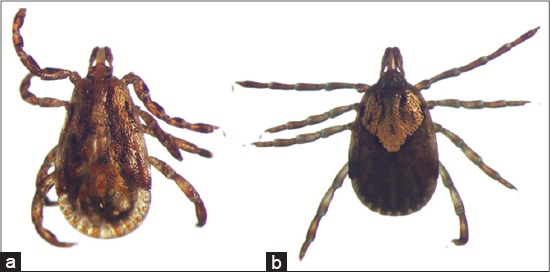
(a) *Amblyomma aureolatum* ♂ and (b) *Amblyomma aureolatum* ♀. Courtesy of USP/Marcelo B. Labruna.

Spotted fever is one of the most important rickettsiosis, caused by an alpha-proteobacteria *R. rickettsii* (etiological agent of the Brazilian spotted fever in Brazil and Rocky Mountain spotted fever in the USA), belonging to the Family Rickettsiaceae, Order Rickettsiales, with features of Gram-negative bacteria, and an obligate association with eukaryote cells and multiplication almost exclusively within endothelial cells, being transmitted to human through the parasitism of ticks [59]. In the last decades Spotted Fever has been reported in continents as North America (USA and Mexico), South America (Brazil, Colombia and Argentina) and Central America (Costa Rica and Panama), always occurring as small outbreaks with more or less severe morbidity and mortality, depending on the time taken for diagnosis and the prompt administration of specific treatment. While RMSF fatality rates are usually 5-10% in the United States, general rates of 20-40% have been reported in Brazil, where untreated cases of Brazilian Spotted Fever (BSF) may present a high fatality rate (80%) but treated cases with specific antibiotics in the 1^st^ days of fever have a reducing of those rates for <10% [60]. In North America, *D. andersoni* and *D. variabilis* are among the most medically important ticks as vector of Rocky Mountain Spotted Fever (RMSF) in the USA and *Amblyomma americanum* (Figure-17), *R. sanguineus* sensu lato and *A. cajennense* complex in Mexico, *A. cajennense* complex in Central America and South America also has been suggested as vectors of *R. rickettsii* [61]. In South America, specifically in Brazil, *A. cajennense* complex and *Amblyomma aureolatum* (Figure-18) are the major vectors of BSF [62]. The mean incubation period of RMSF is 7 days and patients display a diverse range of systemic symptoms with the classic clinical triad of fever, headache, and rash [63]. BSF is associated to severe clinical signals, that generally occur after an incubation period of 5-10 days, with hemorrhagic manifestations and multiorgan dysfunction such as symptoms of high fever, headache, myalgia, malaise, exanthem, vomiting, respiratory distress, petechiae, thrombocytopenia, jaundice, renal impairment, respiratory distress, shock and elevated lethality [64].

Ehrlichiosis is a tick-borne disease caused by obligatory intracellular microorganisms (*E. canis*) that affects white blood cells of domestic dogs infested by *R. sanguineus* sensu lato. *E. canis* develops on the host severe febrile illness characterized by myalgia, depression, reduced white cells and platelets that may lead to bleeding disorders with nosebleeds and anemia. The disease has been diagnosed in all regions of the United States, Europe, Asia and the Americas, including Brazil. The detection is possible by indirect immunofluorescence, enzyme-linked immunosorbent assay (ELISA) or immunoblotting test, which detect the presence of antibodies [65]. Human monocytic ehrlichiosis is caused by *Ehrlichia chaffeensis*, transmitted by tick *A. americanum*, and the white-tailed deer (*Odocoileus virginianus*) is considered the primary reservoir host for this etiologic agent in North America. Human infection by *E. chaffeensis* causes a potentially severe disease characterized by fever, headache, lethargy, myalgia, reduction of sodium levels and platelets and elevated liver enzymes, with fatal outcome at rates of 3% [66].

### Other tick-borne bacterial infections

Tularemia is a contagious disease that affects human, rabbit and other rodents caused by a highly infectious zoonotic agent, the bacteria *Francisella tularensis*. Natural infections have been reported in a range of vertebrates, including mammals, birds, amphibians, fish, and certain invertebrates, with two known cycles of tularemia: the terrestrial and the aquatic, where ticks (*A. americanum*, *D. andersoni*, *D. marginatus*, *D. reticulatus*, *D. variabilis*, *H. concinna* and *I. ricinus*) are the most significant arthropods in the ecology of tularemia, as biological vectors transmitting the pathogen and also important reservoirs [67]. It is a international notifiable disease in Asia, Europe and North America. Two subspecies may cause human illness, *F. tularensis tularensis* (subspecies type A) and *F. tularensis holarctica* (subspecies type B) presenting two forms of clinical manifestation; ulceroglandular tularemia, characterized by an ulcer at the site of the tick bite and enlargement of regional lymph nodes and glandular tularemia, with a regional adenopathy and occurrence of complications by pneumonia and meningitis. Infection is rare among domestic animals, but enzootic outbreaks can occasionally be seen occuring in American and Russian sheeps presenting high mortality during lambing season [68].

Tick-borne relapsing fever is an arthropod-borne spirochetal disease characterized by relapsing fever episodes, transmitted by coxal glands secretions of the soft tick genus *Ornithodoros* in Africa, Asia, Europe and North America and caused by numerous *Borrelia* species with symptoms of relapsing fever include high fever, shaking chills, headache, myalgia, arthralgia, lethargy, and sometimes photophobia, hepatosplenomegaly, lymphadenopathy, petechial rash, facial palsy, myelitis, and radiculopathy. This borreliosis caused by the *Borrelia recurrentis* group with more than 20 species, affect humans, domestic and wild mammals, and also birds, being considered one of the oldest diseases transmitted by arthropods [69].

The emergence of Lyme disease has been associated with the abundance of wildlife hosts (specially the deer *O. virginianus*) and in the prevalence of infection by the causative agent of Lyme borreliosis (*B. burgdorferi* bacterium complex) in nymphal stage of *I. scapularis* tick populations as a result of forest fragmentation in eastern regions of Canada and States in the northeast, midatlantic and upper midwest of United States [70]. It is transmitted by a *I. ricinus* tick complex, primary *I. ricinus*, *I. persulcatus* and *I. hexagonus* in Europe and Asia; and *I. scapularis* and *I. pacificus* in North America [71]. It was identified for the first time in America (in the mid 1970’s), at Old Lyme Town, Connecticut, US, where many acute artritis cases reported in teenagers lead to the clinical characterization [72]. More than 250,000 human cases of Lyme borreliosis were reported from 2000 to 2010 in the United States and the disease is also increasing in Europe, where over 85,000 cases are reported each year in humans. There are about 75 cases per 100,000 inhabitants/year in Europe estimating that more than 10% of tick’s population may be infected [73]. In Brazil Lyme Disease cases have been reported since 1980 in Southeast (Rio de Janeiro, São Paulo States), Mid-West (Mato Grosso do Sul) and North regions (Amazonas), identified by serological tests (ELISA and Western blotting). However, because of serological cross-reactions between different species of *Borrelia*, up to now it was not possible to confirm these human Lyme Disease cases in Brazil characterizing this syndrome as “Lyme disease-Simile.” The Brazilian disease was renamed to Baggio-Yoshinari Syndrome, a new tick-borne zoonosis different from Lyme disease, probably caused by different species of spirochetes or one species modified in the country [74]. Lyme disease is evident in three stages and incubation period lasts from 3 days to a month. The erythema migrans appears in the skin around the tick bite in 50% of cases involving children and in 70% of cases in adults. Additional symptoms include bloating of the local lymph nodes, malaise, fatigue, headache, fever and chills, stiff neck, muscle and joint pain lasting about a month. The disease is rarely fatal, but leads to weakness. The clinical stages comprise a first phase occuring 3-30 days after the tick bite with one reddish rash around the bite. Second stage occurs weeks or months after the initial exposure when bacteria multiply rapidly and invades the circulatory and nervous systems causing arrhythmia and bradycardia, facial paralysis and memory difficulties, respectively. Third phase occurs 1 year after the tick bite with symptoms characterized by intermittent neurological episodes, such as profound fatigue, impaired concentration and loss of short term memory, and also personality changes may occur. Arthritis symptoms are also observed [75,76]. Lyme disease has veterinary importance also affecting dogs, cattle, horses and cats. The most common clinical signs in domestic animals are lameness, loss of appetite, weight loss and kidney disease. In recent studies at the state of Espirito Santo, Brazil, 51% of the canine samples sera tested by indirect ELISA were reactive to *B. burgdorferi*, suggesting that dogs and their ticks can be part of the epidemiological cycle of the causative agent of the Brazilian zoonosis, named Baggio-Yoshinari Syndrome [77].

Q fever is a zoonosis with a worldwide distribution (Africa, Asia, Australia, Europe and North America) caused by *C. burnetii*, a strictly intracellular, Gram-negative bacterium having many species of mammals, birds, and ticks as reservoirs in nature. Humans are often asymptomatic but may manifest an acute disease (self-limited febrile illness, pneumonia, or hepatitis) or a chronic endocarditis, especially in patients with valvulopathy, immunocompromised hosts and pregnant women. Infected ticks (*I. holocyclus*, *Haemaphysalis bispinosa* and *R. sanguineus* sensu lato) are important in maintaining the whole cycle of *C. burnetii*, having a significant role in the transmission among the wild vertebrates (rodents, lagomorphs and wild birds) and domestic dogs [78].

## Tick-Borne Fungi Diseases

Dermatophilosis is a disease where causative agents are fungi (*Dermatophilus congolensis*) transmitted to humans and animals by ticks. It is a exsudative dermatitis that affects primarily ruminants but also humans, characterizing its zoonotic potential. It has a significant economic impact in African and Caribean cattle, and in Australian sheep. A progressive chronical disease related to *A. variegatum* infestation is reported in bovines. In the few known cases in human, the disease has been characterized by pimples and multiple pustules on the hands and forearms, containing a serous exudate that upon rupturing form a reddish crateriform cavity leaving a purplish red scar in 3-14 days. The disease in animals is known as mycotic proliferative dermatitis, being characterized by extensive inflammation with hyperemia and swelling of the affected area and formation of thick scabs of the skin in the bovines, sheep, goats and horses [79].

## Tick-Borne Protozoosis

The Theileriosis is a disease, known as East Coast Fever, caused by the protozoan parasite *Theileria parva* and transmitted by a three-host tick of cattle, *Rhipicephalus appendiculatus*. *Theileria annulata* is causative agent of the Mediterranean or tropical Theileriasis. It is highly pathogenic to cattle and African buffalo, determining a mortality of more than 90% of animals in endemic areas. The characteristic symptom is swelling of the superficial lymph nodes. In cattle, the disease takes about 3 weeks with a pre-patent period of 5-10 days. The acute form is recognized by the high fever, anorexia, tearing, the swelling parotid gland unilateral on the binding site of stage adult ticks, severe dyspnea, the cessation of rumination and a severe loss of general state followed by diarrhea [80].

Babesiosis is a vector-borne disease in North America and Europe and an emerging zoonosis caused by protozoa of the genus *Babesia*, which is transmitted by ixodid ticks. The disease is pathologically characterized by massive destruction of red blood cells and death within a few weeks. Cases of human babesiosis have been diagnosed in North America associated with *Babesia microti* and cases in Europe attributed to *Babesia divergens* [81]. The bovine babesiosis is known as Cattle tick fever (Brazil) and Texas Fever (USA) may be associated with *A. marginale* and *Anaplasma centrale*, being relatively severe for animals introduced in endemic areas who have not had previous exposure [82]. *Rhipicephalus (Boophilus) microplus* and *Rhipicephalus (Boophilus) annulatus* are ticks that economically impact cattle production in tropical and subtropical regions of the world being the vectors of pathogens that cause bovine babesiosis (*B. bovis* and *B. bigemina*) and anaplasmosis (*A. marginale*)[83]. The main symptoms of disease in animals, specifically by *B. bigemina*, are fever, hemolytic anemia, anorexia, lethargy, hemoglobinuria with a high parasitemia, tachycardia, icterus, jaundice and metabolic acidosis. *B. bovis* affects a wide variety of ruminant animals (deer, water buffalo, besides bovine) causing more severe disease often resulting in cerebral babesiosis, characterized by convulsions, hyperaesthesia, and paralysis, leading to death in acutely infected cattle by shock and respiratory distress, with mortality in untreated animals of 50-90% after a incubation period of 8-12 days. The illness is viscerotropic for brain, spleen and liver presenting low parasitemia and thrombus formation in the viscera. Diarrhea, abortions, neurological symptoms (sequestration of parasite in brain capillaries), aggressiveness or extreme apathy, paresis (interruption of movements) and convulsions are others clinical signals of this disease [84,85]. Canine babesiosis is also known as piroplasmosis of dogs and its causative agent is *Babesia canis*. Clinical manifestations are related to the intensity of parasitemia and consist in hemolytic process, progressive anemia, endotoxic shock and coagulation abnormalities. Symptoms are anemia, fever, anorexia, dehydration, weight loss, abdominal pain and renal tenderness on palpation. Strains of *B. canis* present genetic and virulence variability and invertebrate host specificity are the more important: *B. canis* *canis*, *B. canis* *rossi* and *B. canis* *vogeli* with its vectors *D. reticulatus, Haemaphysalis leachi* and *R. sanguineus*, respectively [86].

## Conclusions

Studies on bioecology of ticks, considering information related to their population dynamics to the host and the environment, make possible the application and efficiency of tick control measures and also the prevention programs of vector-borne diseases. The concepts and models of distribution and intensity of tick-borne pathogens are strongly influenced by several factors, such as environmental parameters, changes in land use, changes in human and animal activities. Mainly symptons as fever, headaches, rash, loss of appetite are commom to a vast variety of diseases so scientific comunity as well as the medical community must be aware of the human interference in the ecossystems to be able to identify the possibility of a tick-born disease in time to apply the correct treatment and to interfere in the correct use of the land, specially with the enlargement of cities, taking place of forests and exposing wild hosts to humans and livestock animals. Thus require constant improvements in environmental surveillance to determine levels of infestations and also increasingly high technical standards of professional competence and diagnosis.

## Authors’ Contributions

JBN collected and interpreted published informations for the redaction of this review article. The manuscript was prepared jointly by JBN and KMRD. KMRD and TFM participated in the review process incorporating valuable suggestions for improvement of the manuscript. TFM collaborated with illustrations to the manuscript. All authors read and approved the final manuscript.
